# Segmentation and recognition of breast ultrasound images based on an expanded U-Net

**DOI:** 10.1371/journal.pone.0253202

**Published:** 2021-06-15

**Authors:** Yanjun Guo, Xingguang Duan, Chengyi Wang, Huiqin Guo

**Affiliations:** 1 School of Mechatronical Engineering, Beijing Institute of Technology, Beijing, China; 2 Institute of Remote Sensing and Digital Earth, Chinese Academy of Sciences, Beijing, China; 3 UItrasonic Diagnosis Department, Chengcheng County Hospital, Weinan, Shanxi province, China; Vellore Institute of Technology: VIT University, INDIA

## Abstract

This paper establishes a fully automatic real-time image segmentation and recognition system for breast ultrasound intervention robots. It adopts the basic architecture of a U-shaped convolutional network (U-Net), analyses the actual application scenarios of semantic segmentation of breast ultrasound images, and adds dropout layers to the U-Net architecture to reduce the redundancy in texture details and prevent overfitting. The main innovation of this paper is proposing an expanded training approach to obtain an expanded of U-Net. The output map of the expanded U-Net can retain texture details and edge features of breast tumours. Using the grey-level probability labels to train the U-Net is faster than using ordinary labels. The average Dice coefficient (standard deviation) and the average IOU coefficient (standard deviation) are 90.5% (±0.02) and 82.7% (±0.02), respectively, when using the expanded training approach. The Dice coefficient of the expanded U-Net is 7.6 larger than that of a general U-Net, and the IOU coefficient of the expanded U-Net is 11 larger than that of the general U-Net. The context of breast ultrasound images can be extracted, and texture details and edge features of tumours can be retained by the expanded U-Net. Using an expanded U-Net can quickly and automatically achieve precise segmentation and multi-class recognition of breast ultrasound images.

## Introduction

Breast cancer is the most common malignancy in women and the main cause of cancer deaths among women worldwide [1]. It accounts for 30% of cancer diagnoses in women, with increasing incidence in recent years [2]. According to medical professionals, early detection and therapy of the disease is crucial. Interventional ultrasound, a minimally invasive intervention suitable for breast mass early detection and therapy, is popular due to its avoidance of ionizing radiation, real-time visualization, relatively low price and non-invasive diagnosis [3]. Ultrasound intervention does not involve conventional surgical exposure procedures such as incision and exposure; thus, it requires extremely high operational stability and accuracy, and operators must be able not only to perform the operation but also to read ultrasound images proficiently. However, sonography is much more operator dependent, and reading ultrasound images requires well-trained and experienced radiologists [4]. Fortunately, medical robots have been invented to assist operators in completing ultrasound minimally invasive operations accurately. During a breast biopsy with a medical robot, segmenting the breast ultrasound images into all major functional tissues automatically with high accuracy is of primary importance [5]. With the improvement of computer technology, machine learning and deep learning approaches have been the predominant choices for image segmentation. In recent years, some superior convolutional neural networks have been developed in the field of medical images [6]. Hence, these approaches have been a natural choice for breast ultrasound images. The present study focuses on highlighting the contributions of fully automatic real-time ultrasound image segmentation and recognition techniques for breast ultrasound intervention robots to assist doctors in an operating biopsy presenting an extensive review of the state-of-the-art U-shaped convolutional network (U-Net) [7]. The main innovations of this paper are briefly summarized as follows: the research adopts the basic architecture of a U-Net, analyses the actual application scenarios of semantic segmentation of breast ultrasound images, adds dropout layers to the U-Net architecture to reduce the redundancy in texture details and prevent overfitting [8], and proposes an expanded training approach to obtain the expanded U-Net. It is difficult and expensive to obtain large-scale breast ultrasound images marked by professional doctors, which is far from meeting the needs of large-scale training data. Therefore, this paper uses a data enhancement approach based on geometric transformation to expand the scale of the dataset and solve the problem of overfitting during network training [9].

## Literature survey

To highlight the dire necessity of solving the problem as mentioned in this paper, an explicit literature review was conducted by exploring related studies in the segmentation of breast ultrasound images using state-of-art techniques in the area of convolutional neural networks.

### Segmentation approach for breast ultrasound image

Traditional computer image segmentation methods usually require various manual interventions to improve the accuracy of contours [10]. Surgeons who are not proficient in ultrasound imaging technology hope that breast intervention robots can automatically complete tumour segmentation and recognition [11]. Although various algorithms for breast ultrasound image segmentation continue to be proposed, it is still a difficult task. Breast lesions are usually hypoechoic in ultrasound images with various artefacts, blurred boundaries, low signal-to-noise ratios and uneven intensities, which makes segmentation difficult to automatically perform by computers [1]. Image segmentation methods based on deep learning are applied to various image segmentation tasks [12]. An early popular deep learning for semantic segmentation was patch classification, which is defective because it requires a fixed-size input image and is too slow; at the same time, a large quantity of redundant data is generated [13]. In recent years, convolutional neural networks (CNNs) have successfully completed some computer vision tasks [14]. Later, in 2014, Long et al. [15] proposed a dense prediction network without any fully connected layers, called a fully convolutional network (FCN), in which the fully connected layer was replaced with a convolutional layer and directly output a spatial feature map. FCN is much faster than the patch classification network; however, FCN accepts fixed-size input, its accuracy is not fine enough, context details are not fully extracted, and spatial consistency is lacking. In 2015, Ronneberger et al. [7] made a series of improvements to FCN to propose a symmetric fully convolutional U-shaped convolutional neural network (U-Net), which is more suitable for medical image segmentation. Compared with the general CNN, the input of the U-Net can be of any size, and the output is a heat map with the same size as the input. This end-to-end deep learning is conducive to interdisciplinary applications.

### Improving U-Net architecture for breast ultrasound image segmentation and recognition

U-Net has achieved outstanding results and obtained good segmentation in many major competitions in medical image segmentation, but due to the diversity and complexity of medical images, various improved U-Net algorithms have been continuously proposed for different segmentation [16]. For example, based on the iterative training idea of Auto-Context, Salehi et al. proposed Auto-U-Net [17], which combines the probability map output by the model and the original image into a new input and sends it to the next U-Net in the cascade. Continuous iterative training to continuously improves the segmentation accuracy. However, the number of models required in the Auto-U-Net algorithm is the same as the total number of iterations, which leads to a very large network structure and stack-to-end network models that generate a large number of parameters, but they have not been effectively used. In addition, deep learning-based image segmentation methods (including U-Net) have high requirements for data size and labelling quality, and accurate labelling of medical image data requires very high professionalism. It is very difficult to accurately label medical images in large quantities, which affects the performance of the segmentation model based on deep learning, and it is prone to overfitting and instability during the training process [18]. Moreover, unlike natural images, doctors’ prior knowledge is essential to the analysis of ultrasound images. U-Net draws on the jump connection in FCN and optimizes it. Using cropping and copying in the encoder and connecting to the decoder across layers, the low-feature map and high-feature map are merged to better restore texture details and automatically extract context to achieve accurate pixel-level prediction [19]. Although, context can be captured by U-Net’s encoder, texture details of ultrasound image are rich and highly similar, and in U-Net’s decoder the up-sampling (deconvolution) and fusion layers are followed by a convolutional layer for reducing the number of channels, so the deep texture is redundant and fails in restoring texture details and reduces network efficiency. For example, Yuanyi Zeng et al. [20] proposes a Dense-U-Net to enhance the information flow between layers in the networks. The Dense-U-Net with additional concatenation layers between each pair of convolutional layers which have the same size of outputs to introduce additional large-scale context in semantic segmentation. In response to this problem, this paper directly improves the performance of the model by improving the U-Net network architecture and adjusting the training strategy in a targeted manner. This paper proposes an expanded U-Net, which adds a “dropout” after the fusion layer. That is, in the training phase, a cell in the network is shielded with probability P = 0.5; when the cell’s weights are updated, there is no need to update the shielded cell’s weight [21]. Using dropout technology can solve the problem that texture details of ultrasound images cannot be restored correctly. This paper also proposes an expanded training approach, which introduces the grey-level probability label and its corresponding ternary cross-entropy loss function.

## Materials and methods

### Dataset

A total of 192 clinical breast ultrasound images were used as a training set contributed and annotated by a partner hospital. It included 177 benign tumour images and 23 malignant tumour images; 8 images were used as the test set, including 5 benign tumour images and 3 malignant tumour images. Because this study is retrospective and those ultrasound images that were abandoned during the clinical diagnosis and treatment of patients, it does not interfere with the treatment plan of the patient and does not bring risks to the patient’s physiology; moreover, the images were de-identified before the researchers received them, so the Ethics Committee of Beijing Institute of Technology waived the need for ethics approval and the requirement for informed consent.

**Fig 1:**
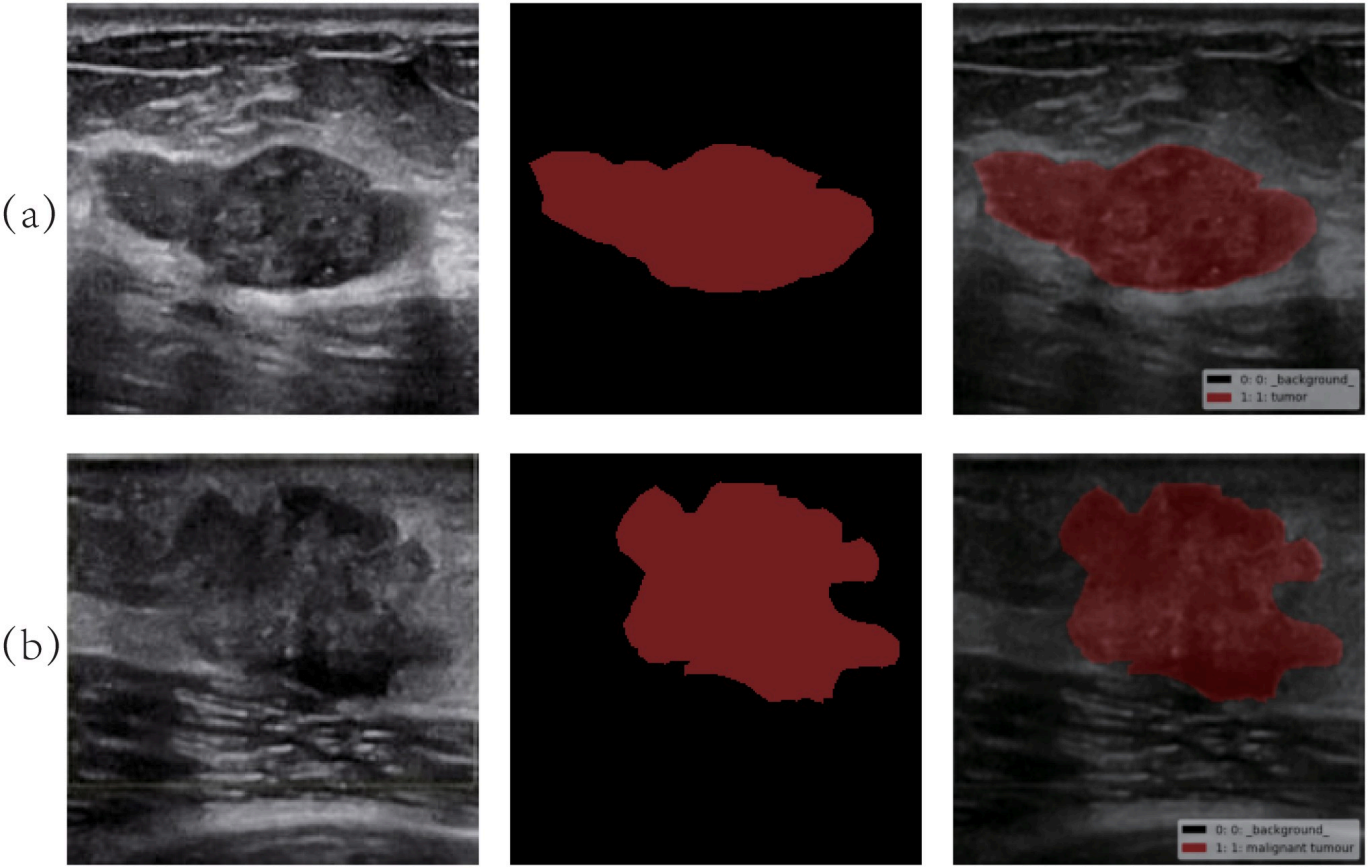
Ultrasound image and label of breast tumour. Picture (a) is a benign tumour, and picture (b) is a malignant tumour.

Ultrasound images were acquired by a LOGIQ P7 2-D B-type ultrasound system and transmitted to an image workstation through a video port. The Keras (2.1.5) framework was applied, and the TensorFlow (1.11.0) backstage was called to implement the segmentation and recognition of breast ultrasound images. The labelling of the tumour area marked by a doctor using the marking tool is shown in Fig 1.

### Expanded U-Net architecture

**Fig 2:**
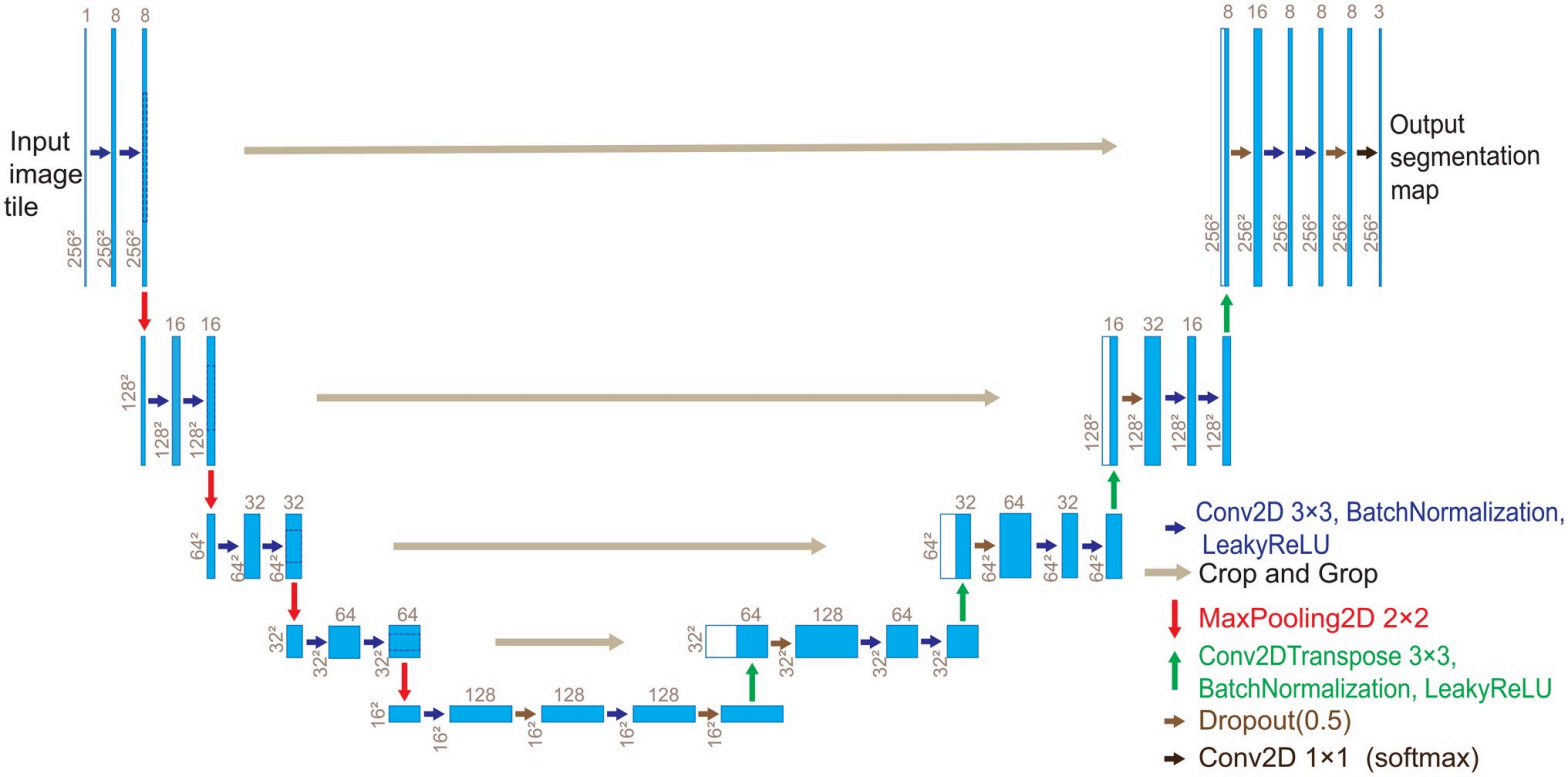
Expanded U-Net architecture.

U-Net contains an encoder on the left and a symmetric decoder on the right, as shown in Fig 2. Both the encoder and the decoder repeat 2 3 × 3 convolutions (padding = “same”). The encoder applies down-sampling (maximum pooling layer) to reduce the spatial dimension of the feature map and then doubles to increase the number of feature channels. The decoder applies up-sampling (deconvolution layer) to gradually restore the details of the feature and its spatial dimensions, while the low-level and high-level feature maps are merged in a cross-layer to form context. In this paper, 7 dropout layers were added to the original U-Net for breast ultrasound image segmentation and recognition. The normalization technique is used after each convolution and deconvolution, and leaky ReLU is used as the activation function. In the last layer, softmax is selected as the activation function to map the 8-channel feature vector to the 3-channel grey-level output map.

### Expanded training approach

The purpose of image segmentation and recognition of the breast intervention robot is to segment and identify the benign, malignant and background areas of the tumour directly and quickly. In the process of network training for U-Net, a new style of labels suitable for automatic segmentation and recognition of breast ultrasound images is proposed, and its loss function (binary cross-entropy function) is expanded to a ternary cross-entropy function [22], forming an integrated end-to-end U-Net expanded training approach.

#### Converting ordinary labels into grey-level probability labels

Three ground truth labels for breast tumour images are obtained using the labelling tool: normal tissue, benign tumour and malignant tumour. First, the ground truth is converted to a three-channel greyscale image with a grey level of 256 and then normalized to grey-level probability labels of 0, 0.5 and 1, as shown in Table 1.

**Table 1:** Label of the expanded U-Net dataset.

Type of label	Ordinary label	Three-channel grey value	Grey-level probability label
**Normal tissue**	0	(0,0,0)	(0,0,0)
**Benign**	1	(127.5,127.5,127.5)	(0.5,0.5,0.5)
**Malignant**	2	(255,255,255)	(1,1,1)

In the output layer of the expanded U-Net, three 1 × 1 convolution filters are arranged to extract the feature vector of the multi-channel feature map, and a three-channel feature map is obtained. Then, a softmax activation function is applied to convert the feature vector into a prediction vector. The prediction vector is assumed to be *P* = (*p*^1^, *p*^2^, *p*^3^); during network training, we can find that *p*^1^ → 0, *p*^2^ → 0.5 and *p*^3^ → 1. These three parameters represent grey-level probability prediction for each pixel on an input image, and each grey-level probability represents a category in the output map of the expansion U-Net. The smaller the difference between them and grey-level probability label, the higher the segmentation accuracy.

#### Loss function

Generally, the U-Net loss function is a binary cross-entropy function, but the expanded U-Net loss function needs to be an expanded cross-entropy function (ECE). “Expanded” has two meanings:

(1) Increasing the loss function update rate

When the training set batch size is N, the information entropy of each pixel is expressed as $E=\sum_{k=1}^{3}y^{\left(k\right)}\cdot logp^{\left(k\right)}$ and $E^{\prime }=\sum_{k=1}^{3}(1-y^{\left(k\right)})\cdot log(1-p^{\left(k\right)})$. *y*^(*k*)^ represents the actual value of each pixel of each channel, *p*^(*k*)^ is the prediction value obtained by the network activation function, and *k* is the number of channels.

The information entropy equation is brought into a binary cross-entropy function to obtain the expanded cross-entropy function, such as Eq (1).

$$\begin{array}{c} DCE(y)=-\frac{1}{N}\sum_{i=1}^{N}E+E^{\prime } \end{array}$$ (1)

where *i* is the mini_batch serial number. When training the network, the pixel prediction value is continuously updated through gradient descent and a backward propagation algorithm.

The output map of the expanded U-Net has 3-channels, as expressed in the formulas for *E* and *E*′. As a result, the update speed of the grey prediction value generated by the expanded cross-entropy loss function is approximately 3 times that generated by the general binary cross-entropy loss function. Without increasing the batch size, the update rate of the loss function is increased by three times. Therefore, the convergence speed of the network training is improved effectively.

(2) Expanding binary cross-entropy function into ternary cross-entropy function

Put the formulas for *E* and *E*′ into Eq (1) to obtain the following:

$$\begin{array}{c} ECE(y)=-\frac{1}{N}\sum_{i=1}^{N}\sum_{k=1}^{3}[y_{i}^{\left(k\right)}\cdot log(p_{i}^{\left(k\right)})+(1-y_{i}^{\left(k\right)})\cdot log(1-p_{i}^{\left(k\right)})] \end{array}$$ (2)

According to the above formula, the grey probability loss of each pixel on each channel is as follows:

$$\begin{array}{c} loss=-y^{\left(k\right)}\cdot logp^{\left(k\right)}-(1-y^{\left(k\right)})log(1-p^{\left(k\right)}),k=1,2,3. \end{array}$$ (3)

During the expansion U-Net training, according to three different values of the grey-level probability label *y*^(*k*)^, three grey-level predictions *p*^(*k*)^ are generated, and the detailed steps are as follows:

1. When the grey-level probability label of some pixel is *y*^(*k*)^ = 1, it belongs to the malignant tumour area, and *loss* = −log *p*^(*k*)^ is obtained from Eq (3). When *loss* → 0, *p*^(*k*)^ → 1 is obtained.
2. When the grey-level probability label of some pixel is *y*^(*k*)^ = 0.5, it belongs to the benign tumour area, and *loss* = −0.5 log *p*^(*k*)^(1 − *p*^(*k*)^) is obtained from Eq (3);
  ∵ *p*^(*k*)^ ∈ [0, 1]

$∴loss\in [-0.5\;\text{log}\;\frac{1}{4},+\infty )$

  When $loss\to -0.5\;\text{log}\;\frac{1}{4}$, *p*^(*k*)^ → 0.5 is obtained.
3. When the grey-level probability label of some pixel is *y*^(*k*)^ = 0, it belongs to the normal tissue area, and *loss* = −log(1 − *p*^(*k*)^) is obtained from Eq (3). When *loss* → 0, *p*^(*k*)^ → 0 is obtained.

Adopting the above algorithm, the binary cross-entropy loss function is extended to the ternary cross-entropy loss function so that the expanded U-Net can automatically output breast tumour segmentation and recognition maps and clearly display the texture details and edge features of breast tumours, which is greater for intuitive vision than ordinary segmentation maps.

## Experiments and results

To verify the feasibility and accuracy of the breast ultrasound image segmentation approach based on an expanded U-Net, the following experiments were carried out in our research, and the experimental results were qualitatively and quantitatively evaluated and analysed.

### Fast convergence of the expanded U-Net training

**Fig 3:**
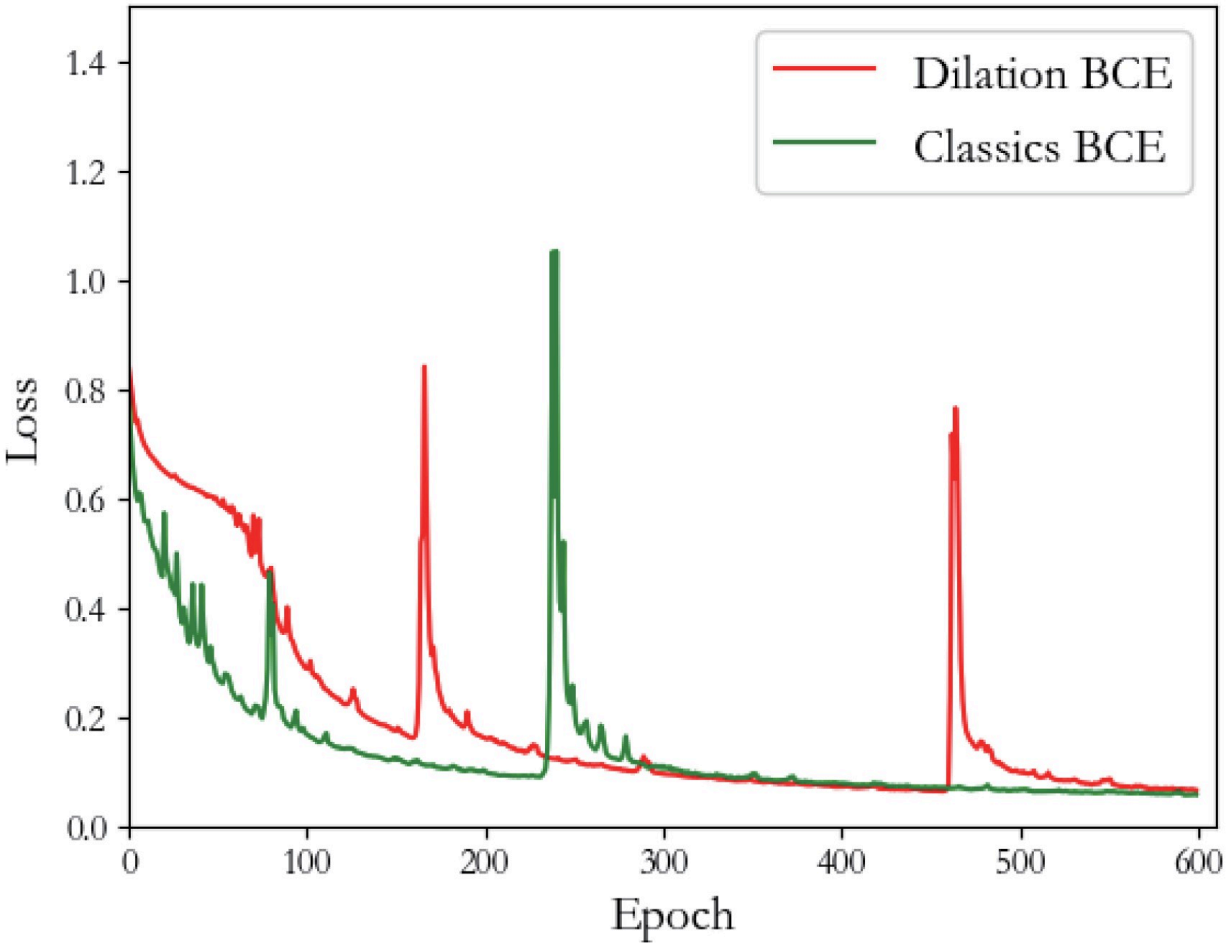
Loss trend in the U-Net training process for the two types of ground truth. The red curve represents the loss trend using the grey-level probability label; the green curve represents the trend using the ordinary ground truth.

To compare different effects on the update rate of weights of expanded U-Net with two different training sets, two ground truth formats are prepared in our experiment. One type is common ground truth, and the labels are 1, 2 and 3. The other type is the grey-level probability label, as shown in Table 1. Both types of ground truth are used to train the expanded U-Net, and the loss trend during training is recorded in Fig 3.

Obviously, when the epoch is 0, the loss of two training iterations is approximately 0.8; as the epoch increases, the difference between the two losses gradually increases. When the epoch is approximately 70, the difference between the two losses reaches a maximum; at this time, the loss of ordinary U-Net is approximately 0.2, and the loss of the expanded U-Net is approximately 0.6. After the two losses are reduced to 0.2, the loss of the former decreases relatively slowly, and U-Net converges when the epoch is approximately 550; the loss of the latter decreases relatively quickly, and expansion U-Net converges after approximately 430 epochs. It can be concluded that using the grey-level probability label to train U-Net is faster than using the ordinary label.

### Validity of changing the loss function from a binary cross-entropy function to a ternary cross-entropy function

The U-Net architecture is end-to-end. The form of the ground truth of the dataset in the input end is changed, so the output end should be changed accordingly. For this purpose, the loss function was redefined, as described in the above section. In this experiment, two U-Net networks were trained. The first used the normal label set to train U-Net with a binary cross-entropy loss function, and the second used the grey-level probability label to train the expanded U-Net with a ternary cross-entropy loss function.

#### Qualitative experimental results

**Fig 4:**
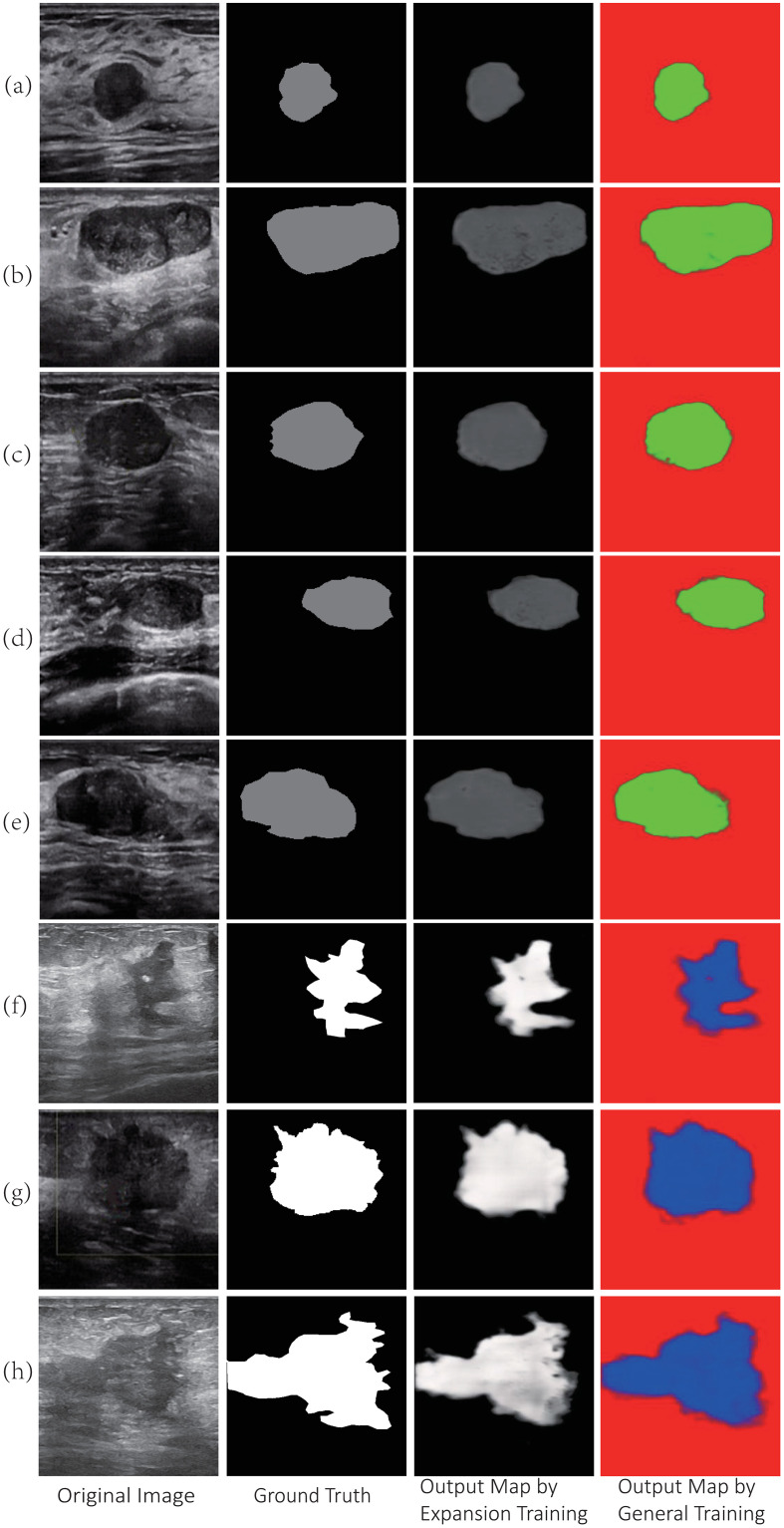
Comparison of test results in breast ultrasound images.

The test set was used to test the two U-Nets, and the test results are shown in Fig 4. The first column shows the original breast tumour ultrasound image, the second column shows the grey-level probability label, the third column shows the test result of the second U-Net, and the fourth column shows the test result of the first U-Net.

In theory, the loss function of the expanded U-Net should be *ECE*(*y*). To make this system compatible with the TensorFlow backend, the calculation of the ternary cross-entropy function proposed in this paper is the same as that of the binary cross-entropy function, so the loss function of the expanded U-Net still calls the optimizer of TensorFlow (the loss function is a binary cross-entropy function). Then, the grey level of benign tumours was close to 2/3 of the grey-level ground truth; the grey level of malignant tumours was close to the grey-level ground truth, as shown in the third column in Fig 4. Comparing the third and fourth columns in Fig 4, it can be seen that the output map of the expanded U-Net retains the texture details and edge features of the breast tumour.

#### Quantitative experiment results

**Fig 5:**
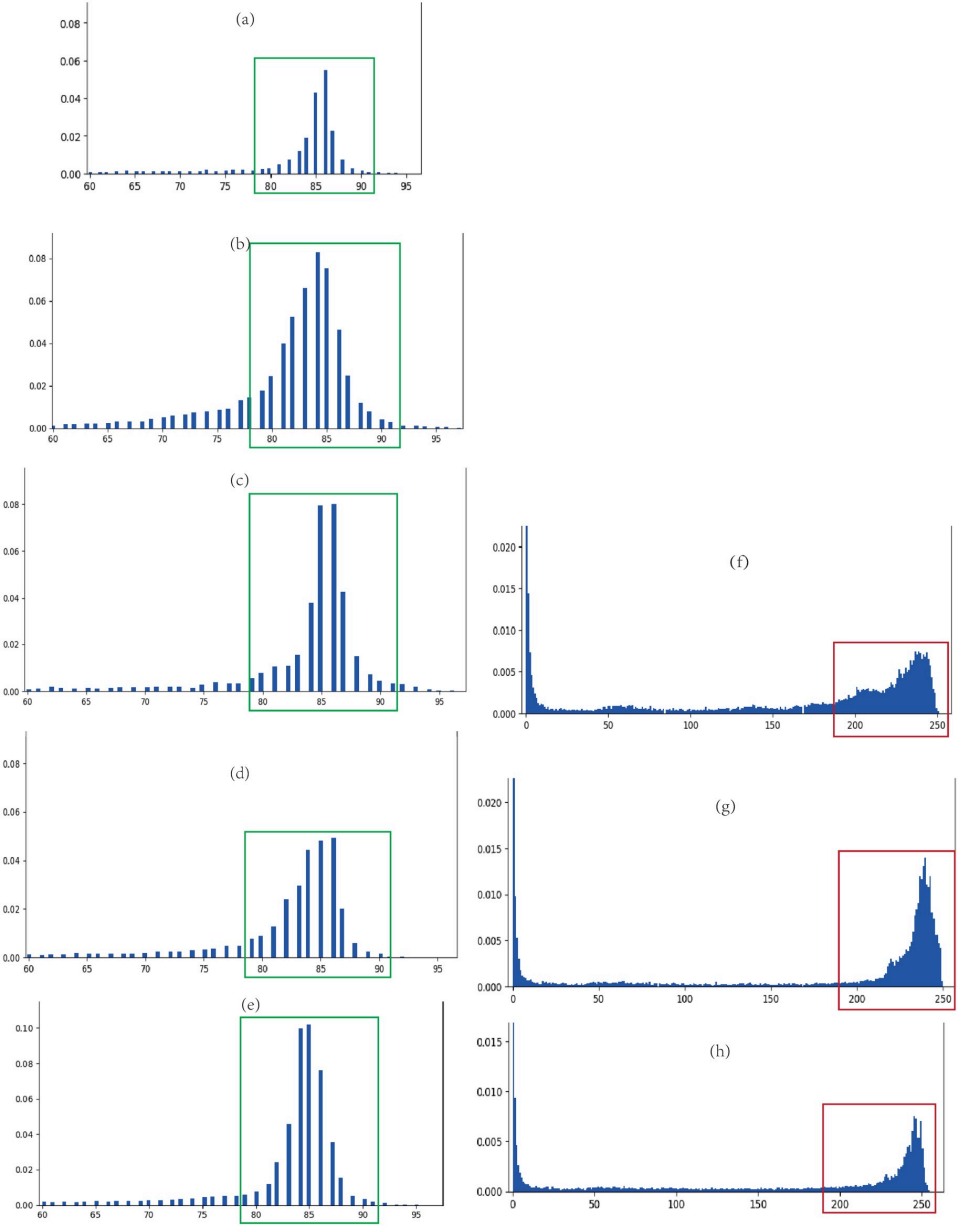
Grey histogram of output map of expanded U-Net. (a) to (e) are the grey histograms of benign tumour, and (f) to (h) are the grey histograms of malignant tumours.

The Dice coefficient and IOU coefficient are commonly used to evaluate the performance of the expanded U-Net [2]. When evaluating a general convolutional neural network, these two coefficients are determined by the overlap between the output map and ground truth. However, grey histograms of the output map in our research are shown in Fig 5. Considering that the output is a greyscale map and the map contains texture details and edge features, it is not reasonable to use the traditional method to calculate Dice coefficient and IOU coefficient.

Fig 5 shows that the grey histogram is affected by the training dataset, and the grey level of the tumour changes within a certain range. The estimation method of the Dice coefficient and IOU coefficient is improved in our research. First, the total grey value (*αx*_*t*_) of the tumour in the ground truth and the grey value *x*_*p*_ of the tumour in the output map are calculated. *α* is the grey reduction rate, the value of benign tumours is *α* ≈ 0.667 and that of malignant tumours is *α* = 1. Assume that the difference in the total grey value between the ground truth and output map is Δ*x* = *αx*_*t*_ − *x*_*p*_, the difference in benign tumours is Δ*x* ∈ [−*δ*, *δ*], and the difference in malignant tumours is Δ*x* ∈ [0, *δ*]. The grey histogram of the output map is shown in Fig 5. The rectangular box indicates the grey of the tumour area. The grey colour of benign tumours is in (80,90), and the grey colour of malignant tumours is in (170,255). Therefore, *δ* = 5 is for benign tumours, and *δ* = 85 is for malignant tumours. Then, the grey difference between the ground truth and predicted output map with a certain grey range is calculated. The geometric meaning of Dice coefficient and IOU coefficient is used to evaluate coefficients suitable for expanded U-Net, which are recorded in Table 2.

**Table 2:** U-Net quantitative analysis results by expanded training and general training.

Type of lesion	Test image	Dice of expanded U-Net(%)	Dice of general U-Net(%)	IOU of expanded U-Net(%)	IOU of general U-Net(%)
**Benign**	(a)	92.0	87.0	85.1	77.0
	(b)	87.5	69.0	77.7	52.6
	(c)	91.6	77.6	84.4	63.4
	(d)	90.4	82.0	82.6	69.5
	(e)	92.7	72.5	86.4	56.8
**Malignant**	(f)	90.6	94.2	82.8	89.0
	(g)	93.2	93.0	87.2	86.9
	(h)	86.2	87.7	75.7	78.0
**Average value**		90.5	82.9	82.7	71.7

The average Dice(standard deviation) is 90.5% (±0.02), and the average IOU (standard deviation) is 82.7% (±0.02) when using the expanded training approach. The average Dice coefficient (standard deviation) and the average IOU coefficient (standard deviation) are 82.9% (±0.02) and 71.7% (±0.02), respectively, when using the general training approach; the Dice coefficient of the expanded U-Net is 7.6 larger than that of the general U-Net; the IOU coefficient of the expanded U-Net is 11.0 larger than that of the general U-Net. Therefore, the expanded U-Net can obtain more accurate segmentation and recognition for breast ultrasound images.

## Conclusion

Through theoretical analysis and experimental research, the paper draws the following conclusions. (1) The context of breast ultrasound images can be extracted, and texture details and edge features of the tumour can be retained by an expanded U-Net; for example, the bright spots inside the malignant tumour are the target in Fig 4. (2) Using the expanded U-Net can quickly and automatically achieve precise segmentation and multi-class recognition of breast ultrasound images. The output map of the expanded U-Net proposed in this paper can clearly show the tumour’s edge features, and the safety and accuracy of interventional surgery assisted by breast intervention robots are improved.

## Supporting information

S1 Data The datasets and the code of the expanded U-Net. The data includes the datasets of training and testing for the expanded U-Net, the code of the expanded U-Net and the results of the experiments.
(ZIP)
