# Supplementary figures and images for: Segmentation and recognition of breast ultrasound images based on an expanded U-Net (part 2 of 2)
Source: PLoS One. 2021 Jun 15;16(6):e0253202. doi: 10.1371/journal.pone.0253202 (PMC8205136; doi:10.1371/journal.pone.0253202)

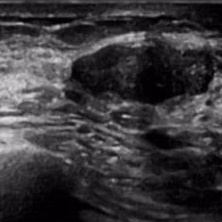

Supplement: S1 Data — The data includes the datasets of training and testing for the expanded U-Net, the code of the expanded U-Net and the results of the experiments. (ZIP) [file pone.0253202.s001.zip › Data/TrainingDataSet/BreastTumourImages/134.jpg]

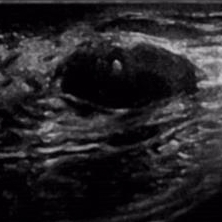

Supplement: S1 Data — The data includes the datasets of training and testing for the expanded U-Net, the code of the expanded U-Net and the results of the experiments. (ZIP) [file pone.0253202.s001.zip › Data/TrainingDataSet/BreastTumourImages/135.jpg]

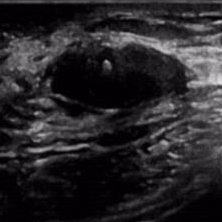

Supplement: S1 Data — The data includes the datasets of training and testing for the expanded U-Net, the code of the expanded U-Net and the results of the experiments. (ZIP) [file pone.0253202.s001.zip › Data/TrainingDataSet/BreastTumourImages/136.jpg]

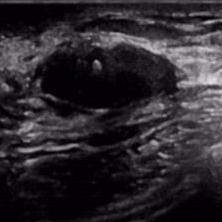

Supplement: S1 Data — The data includes the datasets of training and testing for the expanded U-Net, the code of the expanded U-Net and the results of the experiments. (ZIP) [file pone.0253202.s001.zip › Data/TrainingDataSet/BreastTumourImages/137.jpg]

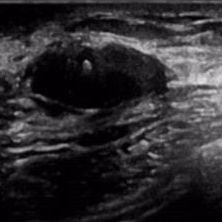

Supplement: S1 Data — The data includes the datasets of training and testing for the expanded U-Net, the code of the expanded U-Net and the results of the experiments. (ZIP) [file pone.0253202.s001.zip › Data/TrainingDataSet/BreastTumourImages/138.jpg]

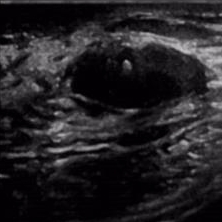

Supplement: S1 Data — The data includes the datasets of training and testing for the expanded U-Net, the code of the expanded U-Net and the results of the experiments. (ZIP) [file pone.0253202.s001.zip › Data/TrainingDataSet/BreastTumourImages/139.jpg]

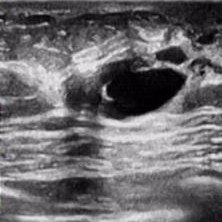

Supplement: S1 Data — The data includes the datasets of training and testing for the expanded U-Net, the code of the expanded U-Net and the results of the experiments. (ZIP) [file pone.0253202.s001.zip › Data/TrainingDataSet/BreastTumourImages/14.jpg]

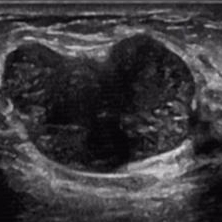

Supplement: S1 Data — The data includes the datasets of training and testing for the expanded U-Net, the code of the expanded U-Net and the results of the experiments. (ZIP) [file pone.0253202.s001.zip › Data/TrainingDataSet/BreastTumourImages/140.jpg]

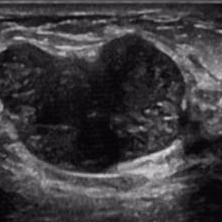

Supplement: S1 Data — The data includes the datasets of training and testing for the expanded U-Net, the code of the expanded U-Net and the results of the experiments. (ZIP) [file pone.0253202.s001.zip › Data/TrainingDataSet/BreastTumourImages/141.jpg]

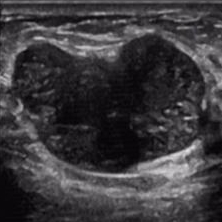

Supplement: S1 Data — The data includes the datasets of training and testing for the expanded U-Net, the code of the expanded U-Net and the results of the experiments. (ZIP) [file pone.0253202.s001.zip › Data/TrainingDataSet/BreastTumourImages/142.jpg]

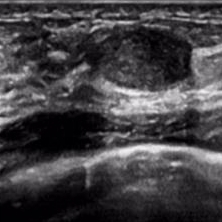

Supplement: S1 Data — The data includes the datasets of training and testing for the expanded U-Net, the code of the expanded U-Net and the results of the experiments. (ZIP) [file pone.0253202.s001.zip › Data/TrainingDataSet/BreastTumourImages/144.jpg]

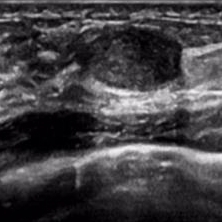

Supplement: S1 Data — The data includes the datasets of training and testing for the expanded U-Net, the code of the expanded U-Net and the results of the experiments. (ZIP) [file pone.0253202.s001.zip › Data/TrainingDataSet/BreastTumourImages/145.jpg]

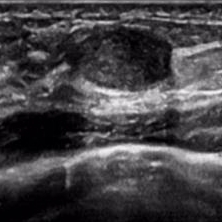

Supplement: S1 Data — The data includes the datasets of training and testing for the expanded U-Net, the code of the expanded U-Net and the results of the experiments. (ZIP) [file pone.0253202.s001.zip › Data/TrainingDataSet/BreastTumourImages/146.jpg]

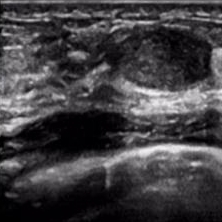

Supplement: S1 Data — The data includes the datasets of training and testing for the expanded U-Net, the code of the expanded U-Net and the results of the experiments. (ZIP) [file pone.0253202.s001.zip › Data/TrainingDataSet/BreastTumourImages/147.jpg]

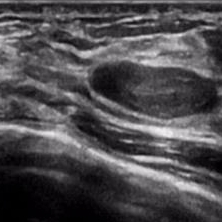

Supplement: S1 Data — The data includes the datasets of training and testing for the expanded U-Net, the code of the expanded U-Net and the results of the experiments. (ZIP) [file pone.0253202.s001.zip › Data/TrainingDataSet/BreastTumourImages/148.jpg]

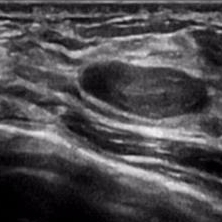

Supplement: S1 Data — The data includes the datasets of training and testing for the expanded U-Net, the code of the expanded U-Net and the results of the experiments. (ZIP) [file pone.0253202.s001.zip › Data/TrainingDataSet/BreastTumourImages/149.jpg]

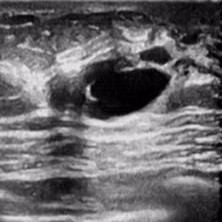

Supplement: S1 Data — The data includes the datasets of training and testing for the expanded U-Net, the code of the expanded U-Net and the results of the experiments. (ZIP) [file pone.0253202.s001.zip › Data/TrainingDataSet/BreastTumourImages/15.jpg]

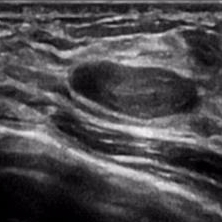

Supplement: S1 Data — The data includes the datasets of training and testing for the expanded U-Net, the code of the expanded U-Net and the results of the experiments. (ZIP) [file pone.0253202.s001.zip › Data/TrainingDataSet/BreastTumourImages/150.jpg]
