# Supplementary material for: Segmentation and recognition of breast ultrasound images based on an expanded U-Net
Source: PLoS One. 2021 Jun 15;16(6):e0253202. doi: 10.1371/journal.pone.0253202 (PMC8205136; doi:10.1371/journal.pone.0253202)
Supplement: S1 Data — The data includes the datasets of training and testing for the expanded U-Net, the code of the expanded U-Net and the results of the experiments. (ZIP) [file pone.0253202.s001.zip › Data/Code/Code_of_Experiment/Quantitative_experiment_results/Experimental_Data_and_Results/1channel_3class_r_and_g_benign/dice.doc]

这是第0张图的dice系数0.8697692564248258

这是第1张图的dice系数0.6896949729881777

这是第2张图的dice系数0.7759112786693562

这是第3张图的dice系数0.8197549620489264

这是第4张图的dice系数0.7248560110848978
