# Supplementary material for: Segmentation and recognition of breast ultrasound images based on an expanded U-Net
Source: PLoS One. 2021 Jun 15;16(6):e0253202. doi: 10.1371/journal.pone.0253202 (PMC8205136; doi:10.1371/journal.pone.0253202)
Supplement: S1 Data — The data includes the datasets of training and testing for the expanded U-Net, the code of the expanded U-Net and the results of the experiments. (ZIP) [file pone.0253202.s001.zip › Data/Code/Code_of_Experiment/Quantitative_experiment_results/Experimental_Data_and_Results/3channel_3class_w_and_b_benign/dice.doc]

a = s2[r][c] - s1[r][c]

if 31.5 < a < 127.5:

这是第0张图的dice系数0.9180125042819298

这是第1张图的dice系数0.9123406250666226

这是第2张图的dice系数0.9240842842414073

这是第3张图的dice系数0.9141474216868842

这是第4张图的dice系数0.916542534257153

a = s2[r][c]*96/127.5 - s1[r][c]

if -50 < a < 50:

这是第0张图的dice系数0.9326805817722986

这是第1张图的dice系数0.9240658290094449

这是第2张图的dice系数0.9347756992449192

这是第3张图的dice系数0.9235795276744718

这是第4张图的dice系数0.9252789415420798

a = s2[r][c]*0.667 - s1[r][c]

if -5 <= a <= 5 :

这是第0张图的dice系数0.9194407871911051

这是第1张图的dice系数0.8745463184616905

这是第2张图的dice系数0.9155037561462488

这是第3张图的dice系数0.904437985097793

这是第4张图的dice系数0.9271651483517077
