# Supplementary material for: Segmentation and recognition of breast ultrasound images based on an expanded U-Net
Source: PLoS One. 2021 Jun 15;16(6):e0253202. doi: 10.1371/journal.pone.0253202 (PMC8205136; doi:10.1371/journal.pone.0253202)
Supplement: S1 Data — The data includes the datasets of training and testing for the expanded U-Net, the code of the expanded U-Net and the results of the experiments. (ZIP) [file pone.0253202.s001.zip › Data/Code/Code_of_Experiment/Quantitative_experiment_results/Experimental_Data_and_Results/3channel_3class_w_and_b_benign/iou.doc]

a = s2[r][c]*96/127.5 - s1[r][c]

if 0<= a <= 127.5:

这是第0张图的iou系数0.8484501973589657

这是第1张图的iou系数0.8388109789634337

这是第2张图的iou系数0.858881667687014

这是第3张图的iou系数0.8418706553213905

这是第4张图的iou系数0.8459423311359503

a = s2[r][c]*96/127.5 - s1[r][c]

if -50<= a <= 50:

这是第0张图的iou系数0.8742044840934582

这是第1张图的iou系数0.8589519942525852

这是第2张图的iou系数0.8776343803223713

这是第3张图的iou系数0.8582926557116161

这是第4张图的iou系数0.861067058868806

a = s2[r][c]*0.667 - s1[r][c]

if -5 <= a <= 5:

这是第0张图的iou系数0.8508934783879496

这是第1张图的iou系数0.777061137928244

这是第2张图的iou系数0.8441742065357566

这是第3张图的iou系数0.8255470459867357

这是第4张图的iou系数0.8642198255651564
