# Supplementary material for: Segmentation and recognition of breast ultrasound images based on an expanded U-Net
Source: PLoS One. 2021 Jun 15;16(6):e0253202. doi: 10.1371/journal.pone.0253202 (PMC8205136; doi:10.1371/journal.pone.0253202)
Supplement: S1 Data — The data includes the datasets of training and testing for the expanded U-Net, the code of the expanded U-Net and the results of the experiments. (ZIP) [file pone.0253202.s001.zip › Data/Code/Code_of_Experiment/Quantitative_experiment_results/Experimental_Data_and_Results/3channel_3class_w_and_b_malignant/dice.doc]

a = s2[r][c] - s1[r][c]

if 0 <= a < 255:

这是第5张图的dice系数0.9549523540843148

这是第6张图的dice系数0.9481354600378378

这是第7张图的dice系数0.8941031347677901

a = s2[r][c] - s1[r][c]

if 0 <= a < 200:

这是第5张图的dice系数0.920222930277333

这是第6张图的dice系数0.9400537868169078

这是第7张图的dice系数0.8890565782458222

a = s2[r][c] - s1[r][c]

if 0 <= a <= 85 :

这是第5张图的dice系数0.9059998894165305

这是第6张图的dice系数0.9315132829567588

这是第7张图的dice系数0.861906919344479
