# Supplementary material for: Segmentation and recognition of breast ultrasound images based on an expanded U-Net
Source: PLoS One. 2021 Jun 15;16(6):e0253202. doi: 10.1371/journal.pone.0253202 (PMC8205136; doi:10.1371/journal.pone.0253202)
Supplement: S1 Data — The data includes the datasets of training and testing for the expanded U-Net, the code of the expanded U-Net and the results of the experiments. (ZIP) [file pone.0253202.s001.zip › Data/Code/Code_of_Experiment/Quantitative_experiment_results/Experimental_Data_and_Results/3channel_3class_w_and_b_malignant/iou.doc]

a = s2[r][c] - s1[r][c]

if 0<= a <= 200:

这是第5张图的iou系数0.8522522068010868

这是第6张图的iou系数0.8868957438486152

这是第7张图的iou系数0.8002992627791077

a = s2[r][c] - s1[r][c]

if 0 <= a <= 85:

这是第5张图的iou系数0.8281533801064502

这是第6张图的iou系数0.871806142367852

这是第7张图的iou系数0.7573255070209515
